# Illegal Passive Smoking at Work 

**DOI:** 10.4061/2011/975678

**Published:** 2011-03-31

**Authors:** François-Xavier Lesage, Frédéric Deschamps, Denisa Jurca

**Affiliations:** Department of Occupational Health, Faculty of Medicine, 51 Rue Cognacq Jay, 51100 Reims, France

## Abstract

*Introduction*. Exposure to passive smoking at work has been forbidden for few years in France. This study's aim is to estimate the prevalence of passive smoking at work (PSW), the characteristics of illegal passive smoking and to identify eventual respiratory effects. *Methods*. Occupational practitioners (OPs) of a French county of 320,000 wage earners were contacted by mail. Then OP answered questions from a standardized questionnaire. These questions concerned the practised job, exposure features linked to PSW and health effects in relationship with second-hand smoke in workplace, and the focus on nonsmoker encountered by OP during the most recent occupational medical examination. *Results*. Ninety-five percent of a total group of 172 OP of Champagne county filled the postal questionnaire. More than 80% of OP's replies identified illegal PSW. The average prevalence of PSW exposure was 0.7% of the total working population. Environmental tobacco smoke (ETS) levels were considered between low and medium for most passive smokers (71%). Main features exposure to ETS at work for non-smokers was associated with female gender (69.5%), age between 40 and 49 years (41.2%) and belonging to tertiary sector (75.6%). Environmental tobacco smoke exposures at work was firstly in the office for 49.7% of the subjects and secondly in the restroom for 18% of them. Main medical symptoms encountered by non-smokers were respiratory tractus irritation (81.7%). Eighty-three percent of OPs indicated solution to eradicate PSW. Illegal PSW is really weaker than fifteen years ago. However, the findings support a real ban on smoking in the workplace in order to protect all workers.

## 1. Introduction

In Europe, fifteen years ago, around 22 million workers are exposed to group 1 of carcinogens [1].

According to International Agency Research Cancer (IARC), the exposed workers had all together 42 million exposures (1.3 mean exposures for each exposed worker). The second most common exposure was environmental tobacco smoke (ETS) (7,5 million workers exposed at least 75% of working time). The World Health Organisation estimates that in the world approximately 700 millions are exposed to second-hand smoke.

Consequently exposure to ETS in the workplace has become a major public and occupational health issue in the recent years [2, 3]. Strong evidence points to ETS as one of the most important contaminants of indoor air and as a major health hazard in the working environment [4, 5].

Exposure of non-smokers to ETS has been associated with increases in risk for a number of diseases, including cancer heart disease and stroke [6, 7]. Moreover passive smoking causes exposure to many potent respiratory irritants. Some studies have found that passive smoking in adulthood increases severity and risk of asthma and respiratory symptoms [8, 9]. Several studies have indicated that involuntary exposure to tobacco smoke for adults results in significant impairment of lung function [10, 11]. In the United Kingdom ETS at work is likely to be responsible for the deaths of more than 2 employed people per working day (617 deaths/year) [12].

The workplace has been established as a major source of exposure to the tobacco smoke. Some occupational groups experience higher levels of ETS than others due to the greater density of smokers at work [13].

A survey of the Police force has found that 80% of those who had never smoked were exposed to passive smoking at work (PSW) [14].

In addition to the time lost because of illness, it has been claimed that smokers are less work productive due to the effects of their habits [15]. Mannino et al. found an excess of days of restricted activity, bed confinement, and work absence for passive smokers [16]. Studies confirm that ETS can reach substantial levels [17, 18].

 Effective prevention of occupational cancer requires knowledge on occurrence of exposure, but information on the real numbers of workers exposed is not always available. Smoking was prohibited in France since 1991. Since 2007, this prohibition covers restaurants as well. The prevalence of daily smoking in France is 30% of men and 22.5% of women aged 17–75 years [19].

 In the occupational health system in France, all the workers have a medical examination by an occupational practitioner (OP) every years or every two years. 

Beyond the medical examination, each OP dedicates one third of his work time for the analysis of the workplace conditions. 

We investigate the prevalence of illegal PSW with regard to age, gender, and job features in a general sample of the French working population in order to assess the potential contribution of ETS exposure to health inequalities and to identify different attitudes towards ETS exposure. The second target of this analysis is to study the effects of PSW on outbreaks of nasal and respiratory symptoms.

## 2. Methodolodgy

Occupational health departments were selected from the area of Champagne county defined by administrative boundaries with a working wage earners population of at least 320,000 individuals. The target population that was defined has employees working in establishments or job sites in the Champagne county (1.3 million inhabitants) employing one or more workers. All commercial and industrial sectors and branches were concerned.

Data were obtained by mailing questionnaires administered to the OP in 2005. Each of them is responsible for occupational health welfare of 3000 workers. The questionnaire was sent to all of them (OP). Firstly, the total number of non-smokers exposed to illegal PSW was quantified. Each OP had to assess the number of persons exposed to PSW among the workers entrusted to them (question: how many workers are exposed to illegal PSW?). Secondly, the questionnaire covered demographic characteristics and position at work. Duration of ETS exposure during working day and levels of ETS was assessed, for non-smoker workers identified by OP during the most recent annual medical examination. 

All the data obtained included the sources, which could be the smoking of colleagues or/and customer. To ascertain passive smoking, OPs obtained data about passive smoking levels has low medium or heavy (question: Was the level of illegal ETS exposure low medium or high?). The questions also addressed locations where the employees smoked at work, in offices, workshops, in areas used for breaks, like coffee-room, or canteens (question: which areas are concerned by illegal PSW: office, workshop, cafeteria, restroom, meeting room, or other?)

OPs were also asked about the existence of chronic respiratory diseases and their nature concerning passive smokers. The following definitions of the dependant variables for respiratory symptoms were used: eye or throat irritation, dyspnea, having cough or bringing up phlegm expectoration, preexisting asthma or asthma worsened by passive smoking or presence of chronic bronchitis. All these specific data concerned the last passive smoker examined by the OPs during the most recent annual medical occupational examination.

The opinion of OPs to enhance respect concerning ban of smoking at workplaces were also asked, as well as solutions suggested by OPs to protect non-smokers against PSW.

Completed questionnaires were placed into envelopes sealed and returned to the research team. Calculation were done using SPSS-PC software.

## 3. Results

A total of 163 OPs answered to the questionnaire, representing about 95% (163/172) of those contacted for the study. More than 80% (131/163) of OP respondant identified, among non-smoking workers entrusted to them, (around 3000 workers by OP) cases of PSW.

Each OPs had assess the number of PSW among the workers entrusted to them. Seventy-two percent of the OPs estimate the prevalence of workers exposed to PSW ranged (0–5%). Adding their assessment, approximately 2200 non-smoking workers were exposed to ETS at work (0.7% of the total population studied of 320, 000 workers). Seventy-six percent of passive smokers were between 30 and 49 years old. Seventy-five percent were nonmanual employees, with a female majority. Medium ETS exposure levels constitute half of PSW situations (Table 1).

Smoking restrictions were reported to exist in all workplaces. Non-smokers indicated that regulations were not obeyed at different locations in their workplace. Second-hand smoke was reported to originate mainly from offices, cafeterias, and restrooms. But PSW in workshops was quite unusual. Seventy-five percent of exposed people were exposed to medium or high ETS levels. 

An association with PSW was found for all respiratory symptoms. Passive exposure to tobacco smoke increased mainly the prevalence of discomforted and irritation of respiratory tractus. Dyspnea, cough, aggravation of pre-existing asthma, headaches, and psychological aggression feeling, concerned only few workers. One third of the population studied complained of respiratory tractus irritation for medium ETS level exposures (Table 2).

Finally 83% of concerned OPs made plans to fight PSW. The recurrent projects of OPs were to enforce the law using letters from the employee union, newspaper advertising, distribution of pamphlets and posters and to improve designated areas for smokers.

## 4. Discussion

The present study was carried out in order to assess the prevalence and the typology of illegal PSW. The main discovery in this investigation on non-smokers adults was that reported PSW was low, but also a reality despite the ban.

Although, the prevalence of PSW has decreased due to legal restrictions on smoking But it is still probably one of the most frequent occupational exposure to a chemical carcinogen in the offices [20].

When occupational exposure to carcinogens in the European Union was estimated from 1990 to 1993, ETS was second, only after ultraviolet radiations (Solar light). About 5% of the employed population was estimated to have been exposed at least 75% of their work time (1). A prevalence of 0.7% of the whole working population exposed to PSW was found in the study, showing the improvement introduced by law for the last 15 years.

Now PSW seems less common at workplace than silica, diesel exhaust, radon, or wood dust. Our results show that compared to circumstances on the international level, exposure to PSW in France is relatively rare.

A first limitation of this study is not to cover self-employed persons: farmers, contractors and so forth, who only represent 15% of the working population. A second limitation is that the study is cross-sectional, and we have no data on the duration of tobacco exposure. Low levels of PSW were marked to allow their inclusion or exclusion since low exposure may have a strong effect on the estimated numbers of exposed subjects. 

The office employees in this study reported the spreading of tobacco smoke to smoke-free areas than did other employees. Non-smokers are usually more sensitive than smokers in detecting the smoke indoor. They also report more frequently discomfort caused by smoking. Besides, there is another limitation in this study which should be taken into account when interpreting the results. We did not include objective measurements of passive smoking, such as salivary, serum or urine cotinine concentration. Relating to this aspect, a study by O'connor et al. compared three methods of ETS exposure measurements on 415 pregnant women: Objectives monitoring of air nicotine, urine cotinine, and employing questionnaire [21]. Women reporting ETS exposure in the questionnaire had a significant verified high level of air nicotine exposure compared with women reporting no exposure, whereas urine cotinine did not differ between these groups. According to S. Jaakkola and K. Jaakkola, the questionnaire method is more successful in accurately approximate air pollutant concentration measurements unlike cotinine concentration measurements [22]. The smell of tobacco smoke was perceptible even through the concentration of nicotine in indoor air was low [23].

Moreover, ETS prevalence is weak: probably less than 1% of workers are concerned. It would have been necessary to process several thousand measurements to provide a representative sample of ETS exposure. Finally, ETS exposure is unlawful. The measurements would have been probably faked, because smokers previously would have ventilated their workplace. But finally it would be preferable to have in this study air nicotine exposure assessment It is one of the lonely surveys to assess the objective prevalence and the effects of PSW by OP. They validated the smoking status of all the workers. In the present study, the interpretation of passive smoking data, does not depend on the validity of self-reported exposure. The OP during one third of their working time, were present in the working area. Moreover the questions answered by OP were simple and clear and the overall quality of the responses was high. The second strength of this study is the systematic nature and the wide coverage, which concerned all commercial and industrial sectors. According to the present questionnaire study, smoking habits and exposure to tobacco smoke varied considerably depending on the position of the employees and the type of the workplace. Moreover it seems that low socio-economic status including mainly females in favour of PSW is confirmed by other authors [24]. In fact, tobacco control should be target on these groups.

In France, a large majority of people spend most of their time inside buildings, and then housing conditions are frequently without the increased ventilation. In our study, PSW was strongly associated with respiratory symptoms. These discoveries rely on those of Leuenberger and colleagues [25].

People with asthma are more sensitive to irritant in the environment than others. All workers exposed to smoke during the course of their work have higher prevalence of respiratory and irritative symptoms. In our study, these results are less unreliable because they are confirmed by OPs. 

Eisner et al. showed that 74% of the workers in taverns and 77% of those employed in bars reported symptoms of respiratory and mucosal irritations [26]. These symptoms disappeared in 59 and 78% of all cases, respectively, after prohibition of smoking in those establishments. These findings provide evidence that non-smoking indoor workers are adversely affected by exposure to PSW, and that underline the importance of workplace smoke-free polices in protecting the health of workers. It is legitimate to breath clean air and to stay healthy.

In our study prevalence of tobacco use at work has been influenced consequently by the tobacco control law, which was enacted in France in 1991 (on workplaces). But voluntary smoking restrictions and designated area for smoking seem insufficient orders to get rid of smoke in the non-smoking areas.

## 5. Conclusion

PSW was common fifteen years ago, and even if worldwide, the ETS is currently common [27], the ETS exposure at work is now quite low. However, roughly 2000 workers are yet currently illegally exposed in our county.

In conclusion, our study has shown that tobacco use and exposure to PSW are still a problem in French workplaces. The results of this research indicate that it may be important to target specific occupations and working populations when planning the work site health initiatives. Neither air conditioning, nor separation of smoking areas can completely clean the air of this significant pollutant. The best protection is not to be exposed to second-hand smoke.

## Figures and Tables

**Table 1:** Demographic characteristics, socioeconomic status, and ETS levels concerning 131 nonsmoker workers exposed to PSW.

		Gender		
		Male *n* = 40 (30.5%)	Female *n* = 91 (69.5%)	Total *n* = 131 (100%)
Age	<19 y	1 (0.8)	—	1 (0.8)
	20–29 y	5 (3.8)	8 (6.1)	13 (9.9)
	30–39 y	14 (10.6)	32 (24.4)	46 (35.1)
	40–49 y	19 (14.5)	35 (26.7)	54 (41.2)
	>50 y	1 (0.8)	15 (11.5)	16 (12.2)
	Age non specified	—	1 (0.8)	1 (0.8) %)

Socio economic status	Non manual employees	28 (21.4)	71 (54.2)	99 (75.6)
	Manual workers	11 (8.4)	10 (7.6)	21 (16)
	Not specified	1 (0.8)	10 (7.6)	11 (8.4) %)

ETS exposure levels	Low	9 (6.9)	18 (13.7)	27 (20.6)
	Medium	21 (16)	45 (34.3)	66 (50.4%)
	High	9 (6.9)	25 (19.1)	34 (26)
	Not specified	1 (0.8)	3 (2.3)	4 (3)

**Table 2:** Medicals symptoms in relationship with socioeconomic status and ETS exposure levels (1).

		Medical symptoms (could be associated for a single worker)				
		Discomfort irritation *n* = 107	Cough *n* = 35	Dyspnoea chronical bronchitis rhinitis *n* = 12	Aggravation of pre-existing asthma *n* = 9	Unpleasant smells headaches psychological aggregations feelings *n* = 29
Socio economic status	Non manual employees *n* = 99	80 (61)	23 (17.5)	9 (6.8)	7 (5.3)	25 (19)
	Manual workers *n* = 21	18 (13.7)	5 (3.8)	—	1 (0.8)	—
	Not specified *n* = 11	9 (6.9)	7 (5.3)	3(2.3)	1 (0.8)	4 (3)

ETS exposure level	Low	22 (16.7)	2 (1.5)		1 (0.7)	7 (5.3)
	Medium	55 (42)	19 (14.5)	5 (3.8)	2 (1.5)	12 (9.1)
	High	23 (17.5)	12 (9.1)	6 (4.6)	6 (4.6)	9 (6.9)
	Not specified	7 (5.3)	2 ( 1.5)	1 (0.7)		1 (0.7)

(1) percentages in boxes (in brackets) correspond to the subgroups of 131 exposed workers with medical symptoms.
